# An Investigation on the Persistence of Uranium Hydride during Storage of Simulant Nuclear Waste Packages

**DOI:** 10.1371/journal.pone.0132284

**Published:** 2015-07-15

**Authors:** C. A. Stitt, N. J. Harker, K. R. Hallam, C. Paraskevoulakos, A. Banos, S. Rennie, J. Jowsey, T. B. Scott

**Affiliations:** 1 Interface Analysis Centre, H. H. Wills Physics Laboratory, University of Bristol, Bristol, United Kingdom; 2 European Synchrotron Radiation Facility, Grenoble, Rhône-Alpes, France; 3 Sellafield Ltd, Seascale, Cumbria, United Kingdom; Belgian Nuclear Research Centre SCK•CEN, BELGIUM

## Abstract

Synchrotron X-rays have been used to study the oxidation of uranium and uranium hydride when encapsulated in grout and stored in de-ionised water for 10 months. Periodic synchrotron X-ray tomography and X-ray powder diffraction have allowed measurement and identification of the arising corrosion products and the rates of corrosion. The oxidation rates of the uranium metal and uranium hydride were slower than empirically derived rates previously reported for each reactant in an anoxic water system, but without encapsulation in grout. This was attributed to the grout acting as a physical barrier limiting the access of oxidising species to the uranium surface. Uranium hydride was observed to persist throughout the 10 month storage period and industrial consequences of this observed persistence are discussed.

## Introduction

Metallic uranium is strongly reactive in the presence of oxidising species. In open air at room temperature it will rapidly form a surface film of hyper stoichiometric UO_2+x_ which, over longer periods, will progressively thicken and form higher oxides at its surface such as U_3_O_8_ and UO_3_ [1,2]. The initial corrosion layer is found to provide an effective physical barrier which limits the rate of ongoing oxidation, slowing it substantially. The relatively large atomic size of uranium in comparison to oxygen dictates that its lattice diffusion in the oxide is very limited and accordingly this controls the way in which new oxide is formed [3]. It is well established that new oxide is formed at the base of the existing layer, meaning that oxidising species must pass through the existing oxide layer in order for this to occur [4,5]. Resultantly, the observed steady-state corrosion rates for uranium in air and oxygen are relatively slow.

In water or water vapour, the observed rates of uranium corrosion are observed to be notably faster and the stoichiometry of the arising oxide also remains closer to a pure UO_2_ [6,7].

$$U^{4+}+2OH^{-}\to UO_{2}+2H^{+}$$ (1)

$$2H^{+}+2e^{-}\to H_{2}$$ (2)

$$2U+3H_{2}\to 2UH_{3}$$ (3)

And/or

$$2U+6H^{+}\to 2UH_{3}$$ (4)

Just as for aqueous corrosion of any other metal, the arising by-product of the water-uranium reaction is hydrogen (Eq 1). If a sufficient concentration of hydrogen accumulates then the corrosion of uranium may switch, whereby the hydrogen released as gas (Eq 2) permeates the oxide barrier, reacting directly with the metal to form uranium hydride (UH_3_)(Eq 3). UH_3_ formation may also be considered as an integral step of the U + H_2_O reaction (Eq 4), however the exact mechanism of the U + H_2_O reaction is still highly debated in the literature [4,6–8]. This product is recognised for its pyrophoric properties and has been attributed to a number of previously documented thermal excursions at nuclear waste facilities [1,9]. Consequently, its potential presence and persistence in any nuclear storage scenario is regarded as highly undesirable because it presents an additional risk factor for the safe management of the waste.

The pyrophoricity of UH_3_ arises from its vigorous and strongly exothermic reaction with oxygen (in air) to form UO_2_ [10]. Accordingly, this observed reactivity has led to the assumption that the hydride, due to its extreme reactivity in air, cannot persist for any significant period in a storage environment at ambient temperatures; wet or dry.

If this assumption is correct then the long term safety risk posed by hydride is significantly decreased as its existence is both transient and brief. For sites like Sellafield in the UK, where substantial quantities of uranium are stored in a range of different environments this is potentially important. Most notably, the redundant legacy ponds and silo facilities at Sellafield, made up of the pile fuel storage pond, pile fuel cladding silo, first generation Magnox storage pond and Magnox Swarf storage silo, all contain uranium in varying quantities in different states and stages of corrosion and are about to undergo decommissioning involving material retrieval and repackaging [11].

As the planned start for decommissioning draws closer, there is a requirement to provide an experimental verification of whether uranium hydride, formed in a waste storage environment, can persist for any significant period.

The current work addresses this challenge by providing a time resolved observation of uranium hydride deliberately formed on grout encapsulated uranium and stored in water for a period of up to 10 months. Previous studies regarding the persistence of uranium hydride in anoxic distilled water [7,12], have observed that for masses of UH_3_ powder below 25g UH_3_ can persist for over 2 weeks. Above this mass, the UH_3_ reaction with water, described by the equation:

UH3(s)+2H2O(l)→UO2(s)+72H2(g) (5)

is reported to be sufficiently heat generating to initiate a near immediate pyrophoric (auto-ignition) reaction. However, in the same study it was determined that in small quantities Eq 5 only partially completes. At 25°C, the reaction was described as initially rapid which then significantly slowed, such that after 2 weeks only 15–20% of the UH_3_ had reacted [7]. In the same study, conditions of 100% relative humidity and 100°C exhibited the same initial rapid reaction and decrease in reaction rate; however, after 40–50 hours, the evolution of hydrogen appeared to cease after 83% conversion of UH_3_ to UO_2_. Complete reaction was then only observed after a reduction in water vapour pressure [7]. The reaction can therefore be described as para-linear, much like the U + H_2_O reaction, and dependent on temperature, sample mass and oxide thickness.

The system investigated here was selected to mimic submersed grouted waste in which uranium metal is an intrinsic reactive component carried over from the imperfect decladding process of Magnox fuel cans. Using 5 mm^3^ samples of uranium in grout we have used synchrotron x-rays to provide direct in situ observation of the hydride growths using diffraction and tomography to determine UH_3_ location, volume and persistence at the micro scale. This experiment is viewed as a precursor for consideration of the potential impact and implications on bulk stored material. The sample used in the following experiment has previously been examined and analysed in [13]. This previous article demonstrated the profound use of synchrotron x-rays to examine uranium encapsulated in grout without the need for breaking the grout confinement. Furthermore, it discusses the effect of grout on uranium hydride formation, determining that even with grout present, hydride growth was undeterred and in some instances, the grout encouraged hydride growth in isolated spots. This behaviour is an important precursory observation for the present article.

## Materials and Methods

A Magnox sourced uranium coupon was mechanically polished using sequentially finer grades of abrasive SiC-paper down to a p2500 SiC grit finish and then a sample 20 x 0.5 x 0.5 mm in size was cut. The sample size was selected to allow sufficient x-ray transmission for analysis. The sample was treated in 5 M HNO_3_ for 3 hours prior to grout-encapsulation to remove the surface oxide, then subsequently rinsed with water and cleaned for five minutes in an ultrasonic bath with ultrapure acetone and then methanol. After this procedure the sample had visibly tarnished, demonstrative of the formation of a thin oxide layer on the sample surface. This oxide was left to grow in air for a further 15 minutes prior to encapsulation to ensure that the oxide had reached the linear rate stage of growth and a complete coherent oxide had been formed, thereby ensuring that all subsequent reactions with the metal had to occur via this interface [6]. The sample was then encapsulated and cured for 3 days in a moist atmosphere using a 3:1 grout mixture of Blast Furnace Slag (BFS) and Ordinary Portland Cement (OPC) and prepared with 0.4 w/c. Further curing was allowed for a week in laboratory atmospheric conditions. Subsequently, the sample was degassed under vacuum (5 x 10^-9^ bar) in a gas rig for 8 hours at 80°C and then heated for a further 16 hours at 170°C to dry the grout before being exposed to a fixed volume of H_2_ at 0.6 bar, conditions expected to form a pure β-UH_3_. After a recorded H_2_ pressure drop equivalent to 0.012 mmol H_2_ uptake, (~3.5% of the total uranium mass transformed to uranium hydride), the reaction was halted by evacuating all H_2_ from the system and cooled under vacuum. The sample was then analysed on the Joint Engineering, Environmental and Processing beam line (I12), Diamond Light Source Limited, UK at this initial stage and then twice more after submersion in the same 40 ml of de-ionised water for 3 and then 10 months respectively, in a semi-sealed environment. X-ray tomography (XRT) and X-ray Powder diffraction (XRPD) were used to respectively image the sample corrosion and crystallographically identify the UH_3_ corrosion products at each consecutive stage. To abide with safety rules during transportation of samples, before each excursion to Diamond Light Source, the sample was heated to 60°C overnight under vacuum to remove excess surface and pore water. This treatment step was expected to cause a brief acceleration in the uranium and UH_3_ corrosion rates. For the first two tomography examinations, the high resolution PCO pco.4000 imaging detector with its Module 4 camera was used with the monochromatic beam to obtain the best resolution possible (1 pixel = 0.98 x 0.98 μm). Due to an upgrade on the I12 beam line, a new high speed imaging detector, a Vision Research Phantom v7.3, was used for the 10 month examination, which had a slightly reduced resolution of 1 pixel = 1.3 x 1.3 μm with the Module 4 camera. It must also be noted that during this particular beam time, Diamond Light Source had a reduced beam current of 136 mA, approximately half of that used in the two previous examinations, and hence the quality of the tomography was slightly reduced. Avizo was used to produce 3D visualisations of the tomography. For the purpose of data reduction, calibration of the detector and x-ray beam energy was determined from diffraction patterns of a cerium dioxide (CeO_2_) standard (NIST—Standard Reference Material 674b) recorded at multiple detector positions [14]. For the initial examination of the sample, the XRPD x-ray energy (114.08 keV) was lower than chosen for XRT (115.97 keV) as for this experiment the resolutions of the diffraction peaks were better at this lower energy, and the XRT tomographs were clearer at the higher energy. However, due to the ongoing development of the beam line the same conditions were not possible to replicate for later examinations, and hence energies of 113.32 and 115.23 keV were used for both measurements for the 3 and 10 month analysis respectively. 2D XRPD data were recorded using a flat panel Pixium RF4343 (Thales) in high resolution mode (2880 x 2881 pixels). This detector has a pixel size of 148 x 148μm and beam size of ~340 x 340 μm

## Results

**Fig 1:**
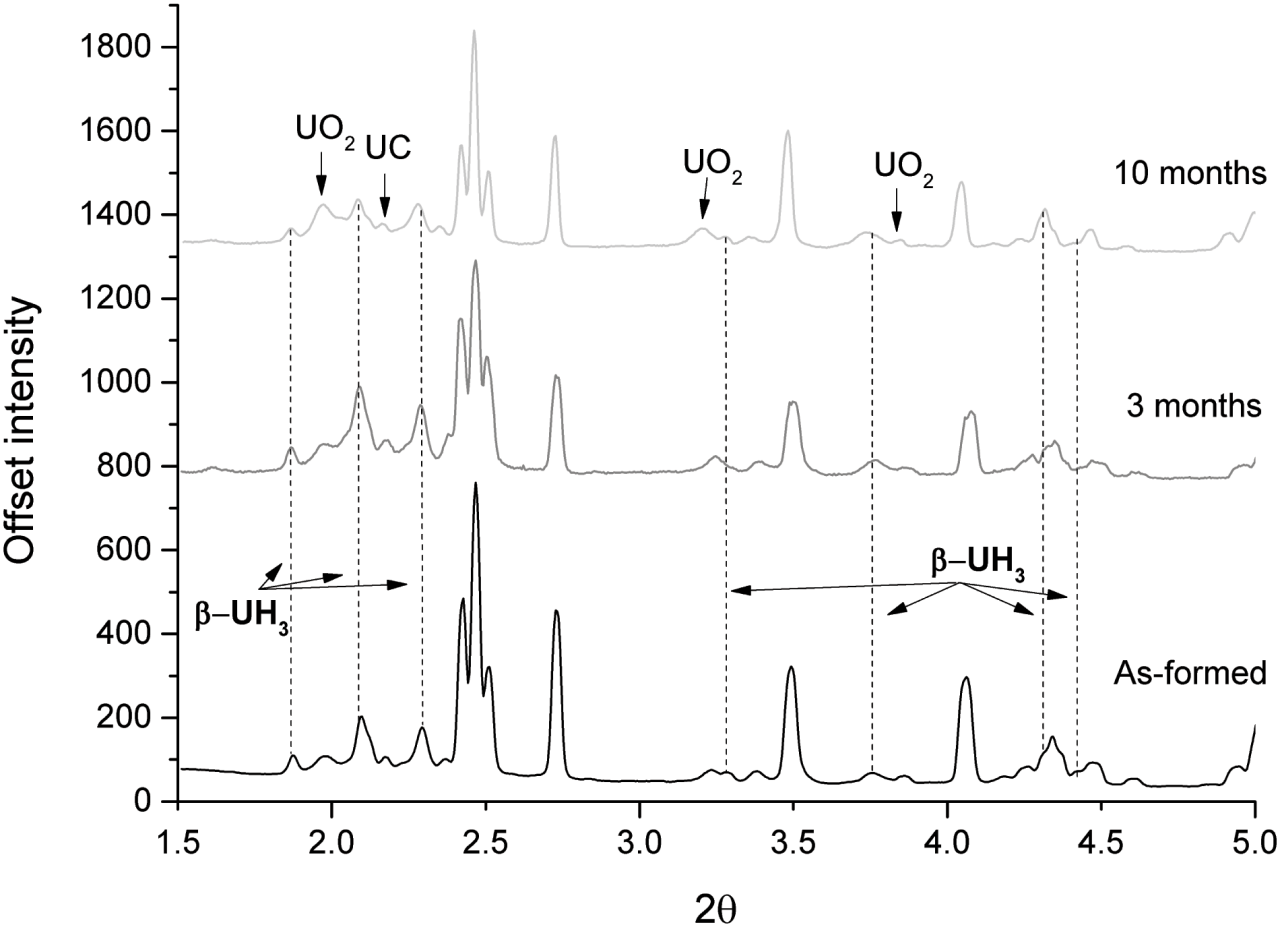
XRPD results of hydrided uranium, encapsulated in cement after 0, 3 and 10 month submersion in de-ionised water. The data was normalised to the first UO_2_ peak, as this was considered the least strained constituent of the sample. Intensities of peaks at each time period vary due to imperfect beam line set up at each occasion. All unlabelled peaks are ascribed to uranium.

XRPD results of the three sample examination periods are displayed in Fig 1. Due to time constraints, it was only possible to perform two line scans across the width of sample (0.5 mm) at different heights for each examination. Furthermore, the line scans were not performed at the exact same positions for each examination owing to difficulties in exactly repositioning the sample. Therefore it would be unrealistic to compare the ratios of oxide to hydride and hence growth of the respective corrosion products in this case, as these line scans would not represent the bulk of the sample. Nevertheless, Fig 1 clearly indicates that β-uranium hydride (UH_3_) is present on the surface of grout encapsulated uranium. A peak ascribed to UC reflects the high impurity content of Magnox uranium [15,16].

**Fig 2:**
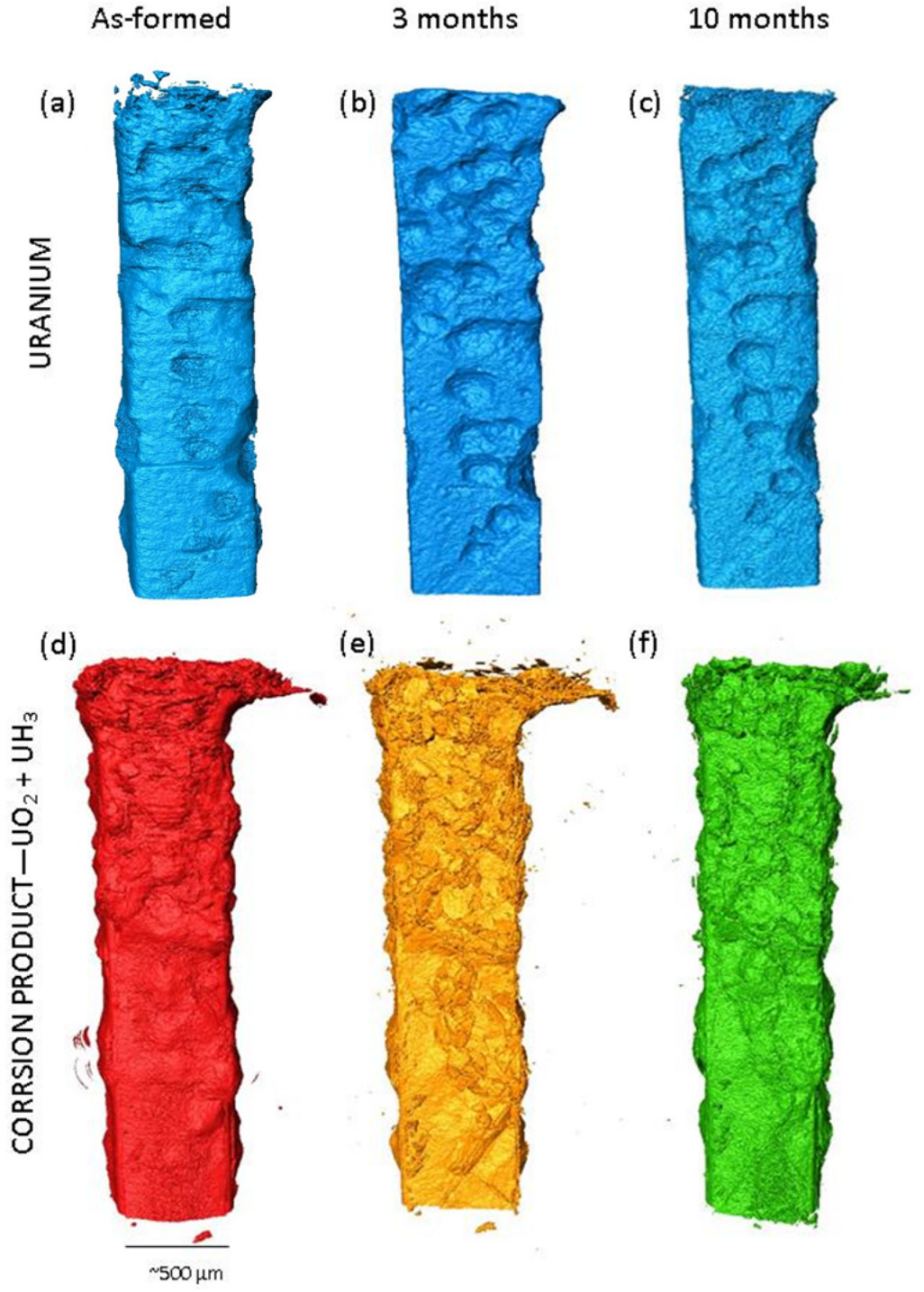
Three dimensional representations of the uranium sample encapsulated in cement over the 10 month period. Images (a) to (c) display what is left of the uranium inner core after initial hydriding (a) to 10 month submersion in water (c). Images (d) to (f) show the corresponding corrosion layers associated with each time period (0 to 10 months).

Fig 2 displays 3D visualisations of the uranium sample before and after 3 and 10 months submersion in water (a more thorough description of the sample before water submersion can be found in [13]). The encasing grout has been purposefully removed from the reconstructions for ease of uranium analysis. As the pre water submersion tomography (Fig 2(a) and 2(d)) was performed above the uranium K absorption edge, image quality was affected by enhanced absorption of the uranium. Consequently, the greyscale representing the uranium metal overlapped that of the corrosion products in some places and measurements extracted from this data set were only taken when the contrast between the uranium and corrosion products was deemed satisfactory. This issue was resolved in the latter two inspections by performing tomography below the uranium K absorption edge.

**Fig 3:**
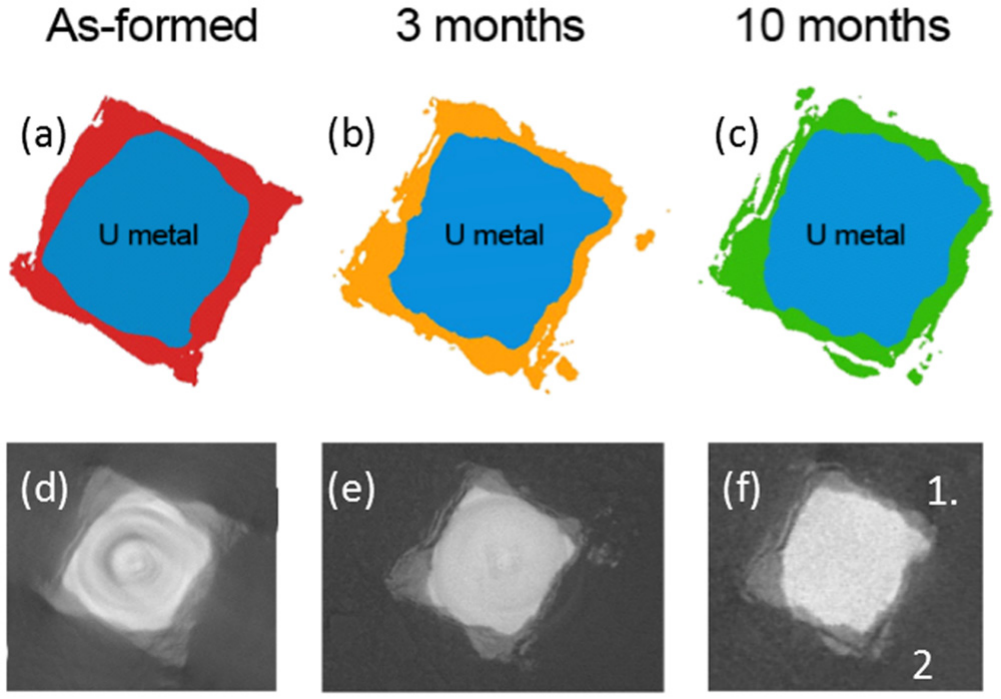
Cross sections at the same position of the uranium encapsulated in grout over time. Images (a) to (c) are accurate representations of the uranium (blue) and surrounding corrosion products (red, orange and green) perpendicular to the length of the sample. Images (d) to (f) are radiographs corresponding to the above representations, but with the additional surrounding grout.

As identified in [13], on first inspection, two morphologies of corrosion products were identified on the sample at each stage: i) deep pits in the metal filled with blisters of less dense corrosion product that was flaky and porous at the surface, characteristic of UH_3_ (Fig 2(a)–2(c); and ii) an enveloping layer blanketing the remaining surface of the metal attributed to surface oxide; UO_2_. The dimensions of 12 identified pits were determined and the average thicknesses of the sites were ~85 μm, with diameters as large as ~340 μm (Table A in S1 Table). Over the 10 month period submersed in water, no significant changes in the overall corrosion layer could be identified; however, measurements of the corrosion layer suggest a volume increase of 4.5% average ±3% between analysis at 3 and 10 months (Table B in S1 Table). Using Avizo software, the total volume of corrosion products calculated after 3 months was ~0.242 mm^3^ (34.2% of total sample volume measured) and, over the following 7 month period, the corrosion products increased to 0.282 mm^3^ (38.7% of total sample volume measured). A volume increase would be expected from the corrosion of uranium to either UO_2_ or UH_3_, as these corrosion products are almost half the density of the metal [17]. Further measurements of the oxide layer at 70 random locations on all three tomography reconstructions away from hydride blisters revealed an average oxide thickness of 10.1, 12.8 and 13.9 μm for the as-formed, 3 and 10 month reconstructions respectively indicating a 2.7 and 1.1 μm growth on the surface of the uranium between each time period (Table C in S1 Table). However, the average oxide thickness for each time period had a range of ^+^/_-_ ~11 μm, demonstrating the significant inhomogeneity of the oxide layer. These measurements were made exclusively from the ubiquitous layer covering the surface of the metal, as the slight density difference (and therefore tomograph greyscale) between the UH_3_ and UO_2_ was not sufficiently distinct to differentiate between the two corrosion products at the hydride sites (e.g. in Fig 3(d)). Considering the expected corrosion products of water exposure to uranium (Eq 1), and the measurements obtained from tomography, the extra 4% gain in corrosion products directly determined from our measurements is therefore most reliably ascribed to UO_2_ growth, perhaps with limited UH_3_ growth owing to the disputed mechanism of the U + H_2_O reaction (Eq 4), although no morphological evidence was observed for additional UH_3_ formation. This is supported by the plated morphology of the corrosion surface exhibited in Fig 2(e), Fig 3(e) and 3(f), an indicative feature of surface oxide formation which starts to exhibit spallation at thicknesses >2 μm, due to in-plane compressional stresses generated during formation.

Fig 3 shows a sequence of tomography slices of the same position for each time period, and showing that despite a growing corrosion layer, some corrosion product identified as hydride on the basis of blister like morphology (labelled 1 and 2 on Fig 3(f)), exhibited some migration into a large pore within the grout. Fractures parallel to the metal surface were also revealed within hydride blisters, highlighting the friable nature of the material (e.g. hydride site 2 in Fig 3(f)). Fragility was not observed in hydride blister sites tightly bound to the encasing grout.

## Discussion

The results obtained from this experiment provide strong evidence that β-hydride artificially formed on grout encapsulated uranium will persist during storage in water for a period of 10 months at room temperature. This observation is consistent with previous studies which have used more indirect means of demonstrating the persistence of uranium hydride in distilled water, as reviewed by Haschke (1995), Baker et al., (1966) and Newton et al., (1949) [7,12,18]. The results also confirm that under the conditions studied uranium hydride is less susceptible to rapid and continued oxidation (and perhaps pyphoricity) in a grouted system than in air.

### Oxidation of U metal

**Fig 4:**
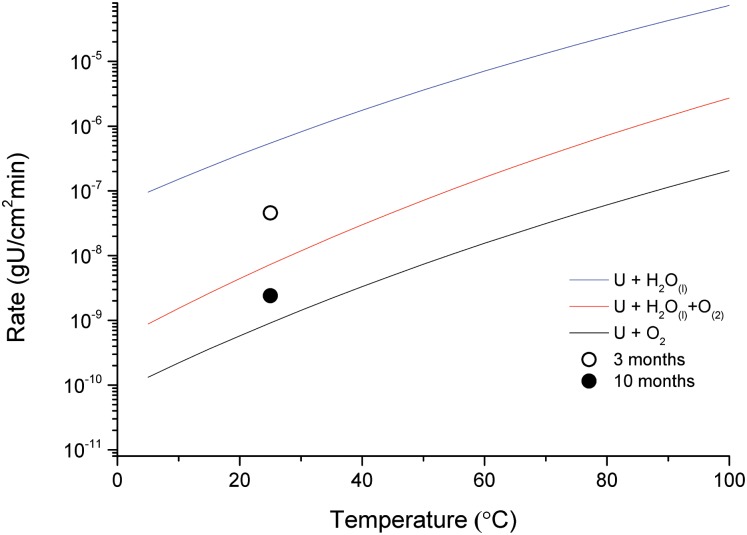
A comparison of the measured oxidation rates (R) to the empirically derived models for the U + 2H_2_O_(l)_→ UO_2_ + 2H_2_, U + 2H_2_O_(l)_ + O_2(g)_→ UO_2_ + 2H_2_O and U + O_2(g)_→ UO_2_ systems provided by Delegard and Schmidt, Ritchie and Haschke respectively [5,12,19]. Results collected from the initial investigation [13] are also presented; Bare U (i.e. no grout) and U + grout (i.e. after 1 week encapsulated in grout).

Fig 4 provides a comparison of the uranium oxidation rates measured here (assuming linear rate kinetics and equal oxide growth rates across the surface of the metal) against those provided empirically by Delegard and Schmidt (2009) [19] for the U + H_2_O_(l)_ reaction at temperatures 10–350°C, Ritchie (1981) [20], for the U + H_2_O_(l)_ + O_2_ reaction from 25–100°C and Haschke (1995) [6] for the U + O_2(g)_ regime up to 200°C. The rate equations were log_10_(60000R) = 9.9752-(3564.8/T), log_10_(60000R) = 9.466-(3836/T) and lnR = 6.192-(8077/T) for each environment respectively, where T is temperature in Kelvin and the rate (R) = gU/cm^2^.min [6,19,20].

In the first week after grout encapsulation, it was expected, based on the previous investigation in [13], that the uranium would initially corrode following the U + H_2_O_(g)_ reaction, forming a product of stoichiometric UO_2.00_ (labelled as U + grout in Fig 4). However, during subsequent artificial UH_3_ formation, oxidation rates were expected to have briefly accelerated as the sample was heated prior to and during hydride formation. The sample was again heated (under vacuum) before each further synchrotron examination which may have further tampered the oxidation rates, again by briefly accelerating corrosion. However, on submersion in water at 25°C, oxide growth rates derived from the previously observed differences in oxide thicknesses between time periods (2.7 and 1.1 μm growth) show an appreciable reduction in oxidation rate from the pure unrestricted U + H_2_O_(l)_ regime. This observation was not attributed to an increase in oxygen levels as the rate equations may suggest. This is because grout mixtures provide an effective barrier against water diffusion and also BFS typically creates highly reducing conditions and hence oxygen will be rapidly scavenged prior to reaching the uranium [21]. To confirm this, an additional sample prepared exactly as the featured sample but excluding the hydriding procedure (i.e. no heating or exposure to hydrogen) was submersed in de-ionised water for ~12 months and the water was periodically measured for its oxidising potential. The Eh (reduction potential) was measured as -100 mV, indicating that a reducing environment was present within the grout (likely lower than -100 mV). In a stagnant reducing water environment, diffusion is the only transport method for oxidising species and, consequently, it is proposed that the lower than expected oxidation rates determined for the uranium metal were due to the dense structure of the grout, physically limiting the access of the diffusing oxidising species to reach the surface of the metal. This would explain the range in oxide thickness observed across the uranium surface (+/-11 μm), which would have resulted from different diffusion rates achieved through the grout at different points across the surface of the metal. BFS grout mixtures matching the formulation used here, have been chosen purposefully for their low gas and liquid permeability in the nuclear waste industry [22].

### Oxidation of UH_3_

Considering the influence exerted by the grout on uranium metal oxidation, it would be expected that the same influence would exist for the oxidation of UH_3_ in grout. Diffusion of the oxidising species to the UH_3_ is limited by the dense structure of the grout, which subsequently would also prevent diffusion of the outgoing corrosion products for both the uranium and UH_3_ oxidation too, i.e. generated hydrogen would be locally confined at the uranium-grout interface (Eqs 1–5). For every mole of UO_2_ that is created by UH_3_ oxidation, 3.5 moles of H_2_ would also be released (Eq 5). Therefore, if hydrogen diffusion away from the UH_3_ is slow and hydrogen can accumulate to sufficient pressures then conditions favourable to further UH_3_ formation develop over prolonged periods of time. No evidence for hydride growths are observed here, but the transition between UH_3_ and UO_2_ would not be marked by a volume difference due to the similarities in density and therefore it is possible that both reactions could be occurring simultaneously or in cycles; however, is more likely that the original hydrides remained unreacted.

Using an average UH_3_ blister diameter of 210 μm measured from 12 well defined hydride sites and approximating the volume of a hydride site to that of a sphere, the volume of UH_3_ expected to transform to UO_2_ over the entire 10 months may be calculated using the rate at 25°C provided by Haschke (1995) for the UH_3_ + H_2_O reaction: 1.6 x 10^-9^ gU/cm^2^min[12]. Assuming the UH_3_ formed here had a similar surface area to that used in Haschke’s experiments (0.5 m^2^/g of UH_3_ powder), over a 10 month period only 0.57% of the UH_3_ would have been expected to react, equating to a covering oxide thickness of ~0.8 μm for each individual hydride site [7]. If the oxidation behaviour of UH_3_ is dependent on its surface area or surface to bulk ratio (as it is for the uranium hydriding reaction [23,24]) then the rate of oxidation is expected to be even slower than predicted here, as powdered UH_3_ would retain a higher surface area than UH_3_ still adhered to the metal. Nevertheless, this calculated oxide thickness is smaller than the resolution of the tomography scans, but the plated morphology indicative of UO_2_ is exhibited after 3 months (Fig 2(e)), suggesting a thicker oxide is present. However, it is expected that an oxide layer was present prior to hydride formation that remained between the grout and UH_3_ interface, which may have only started fracturing or spalling after 3 months following some further oxide growth. During the original grout setting period, a water saturated grout environment provides an abundance of negatively charged mineral sorption surfaces for aqueous uranyl, (produced by oxide dissolution when in contact with the fresh initially oxidising water), to become adhered to [19]. Hydrides forming a layer beneath a pre-existing oxide confined by grout may have subsequently been protected from rapid and significant continued oxidation. Oxidation of UH_3_ in grout would therefore be expected to have a reduced rate in comparison to the predicted rate demonstrated by Haschke [12]. Furthermore, due to the incomplete oxidation of UH_3_ by water (Eq 5) under the constant reaction conditions observed by Baker (1966), Haschke (1995) predicted the formation of an oxide-hydride with a stoichiometry of UO_1.2_H_0.6_ [7,12]. This product was detected at temperatures of 100–150°C and expected to form at 25°C; however, none was observed in the current experiment which further corroborates a reduced reaction rate for UH_3_ encapsulated in grout [12,25].

### Loss of corrosion products into grout

Tomographic reconstructions of the sample over time indicate an increase in corrosion product volume, which was attributed to additional uranium oxide formation over time. However, cross sections of the sample display some apparent loss of corrosion product into the surrounding pore volume (Fig 3). Grout pore waters are renowned for containing strong complexing ligands and previous studies have shown that uranyl cations readily complex with phosphates [26], sulphates [27], hydroxides [27][28] and carbonates [27]. However, for this to occur, the pore water must be oxidising to transform U^3+^ (UH_3_) or U^4+^ (UO_2_) to its higher solubility U^6+^ uranyl form of (UO_2_^2+^) [29]. As stated previously, the grout pore water was measured as reducing and hence any significant dissolution of the uranium is improbable. This ‘break up’ was therefore attributed to vibrations during transportation which consequently damaged the sample, despite care being taken not to cause any significant disturbance. Nevertheless, this observation highlights the importance of correctly sealing uranium containing wastes in the grout, else a tendency for corrosion product particulates to form sludge may occur [12].

### Implications for the nuclear industry

It is recognised that the initial hydride formation conditions used in this investigation were not typical for hydride formation in some ambient pond storage conditions. For example, here the XRPD data indicates β-UH_3_ as the dominant phase of uranium hydride, which is expected from reaction temperatures above 100°C [30]. Conversely, the majority of encapsulated uranium in legacy pond storage is typically below 80°C and, correspondingly, increasing proportions of α-UH_3_ would be expected if hydride formation did occur. Little literature has been published on α-UH_3_ and the oxidation reaction behaviour is not known. Thus experiments using β-UH_3_ as a surrogate for α-UH_3_ are the closest insight to α-UH_3_ reaction behaviour so far. However, comparative data for plutonium hydrides prepared slowly at temperatures below 100°C showed a significantly less stable and finer hydride powder than formed at higher temperatures [30,31]. The corrosion of uranium is often used as a surrogate for plutonium, for example in Dinh et al., 2011 [32]. Consequently, hydride produced in most pond stored nuclear waste may, in fact, be more reactive than predicted herein. As a result, α-UH_3_ oxidation may occur at a greater rate in water, minimising the total residual volume of α-UH_3_ available for subsequent oxidation should air exposure occur later during the lifetime management of the waste.

There are a few instances, however, where conditions conducive to β-UH_3_ formation may occur, such as in submersed silos where temperatures are higher and sludge can accumulate. Oxidant diffusion pathways in silos are at least 100 times that of the sample tested here, and therefore diffusion from oxygenated water would be further limited and anoxic conditions would expectedly prevail for longer at the uranium surface, potentially enabling persistence of UH_3_ over much longer periods if undisturbed. If UH_3_ is present in bulk quantities (>25 g) within grout, which is expected to form over long periods of storage and previously observed behaviour of UH_3_ encapsulated in grout [13], oxidation is expected to initially be faster [10,15]. If the grout was intact, limited diffusion to and from the metal would allow large quantities of UH_3_ to persist. However, in reality the volume expansion caused by UH_3_ and UO_2_ formation would expectedly cause the encasing grout to fracture and disintegrate over time, providing pathways for water (anoxic or oxic) to access the UH_3_ and begin a slow controlled oxidation. The protective oxide layer forming on the surface of the UH_3_ blisters may then dampen the oxidation reaction when exposed to air, even if the inner core of the blister still persists.

It should also be noted that grouted intermediate level waste drums containing uranium, whilst initially formed under saturated conditions, are stored nominally dry. This dictates that water corrosion of encapsulated uranium will only occur for a period of perhaps close to that studied here. However, eventual emplacement in a geological disposal facility will see re-establishment of water corrosion once more.

The next logical step for ongoing research is to understand the location and abundance of the UH_3_ and UO_2_ within the grout system. Neutron tomography or secondary ion mass spectrometry would provide further understanding of the UH_3_ oxidation rates in the current sample, which will be used to produce rates for larger wet storage systems.

## Conclusions

The present work has demonstrated that laboratory made β-UH_3_ will persist on grout encapsulated uranium submersed in a semi sealed volume of water for periods up to 10 months. X-ray diffraction data indicated β-UH_3_ and UO_2_ peaks were present after 3 and 10 months submersion in water. X-ray tomography allowed comparisons of uranium and uranium hydride oxidation rates to determine that the conditions within the grout were likely an anoxic H_2_O system. In addition, it was identified that oxidation was limited by the encasing grout body. This was identified as a legitimate cause for the current and future persistence of hydride in grout.

## Supporting Information

S1 Media Video showing the 3D volume renders of the 10 month versus as-received XRT scans. (MP4)

S1 Render Sub-volumed 4x4x4 radiography data stack for the uranium sample after 10 months water. (ZIP)

S1 Table Tables containing time resolved corrosion volume data and oxide/hydride thickness data. Table A. Dimensions of hydrides observed on the sample surface. As-received measurements were not included in the full analysis owing to the poor quality of the XRT scan. Table B. Volumes of the sample at each reaction stage. As-received measurements were not included in the full analysis owing to the poor quality of the XRT scan. Table C. Oxide thicknesses observed on the sample surface at each reaction stage.
(ZIP)
