# Supplementary figures and images for: An Investigation on the Persistence of Uranium Hydride during Storage of Simulant Nuclear Waste Packages
Source: PLoS One. 2015 Jul 15;10(7):e0132284. doi: 10.1371/journal.pone.0132284 (PMC4503347; doi:10.1371/journal.pone.0132284)

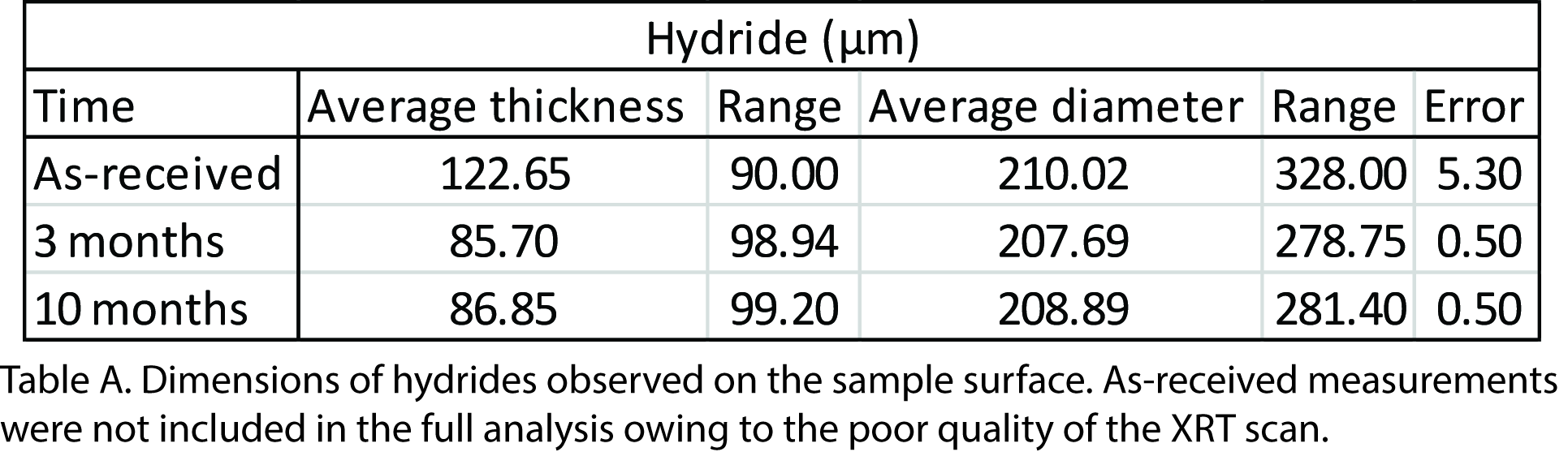

Supplement: S1 Table — Table A. Dimensions of hydrides observed on the sample surface. As-received measurements were not included in the full analysis owing to the poor quality of the XRT scan. Table B. Volumes of the sample at each reaction stage. As-received measurements were not included in the full analysis owing to the poor quality of the XRT scan. Table C. Oxide thicknesses observed on the sample surface at each reaction stage. (ZIP) [file pone.0132284.s003.zip › S1_Table/Table A.tif]

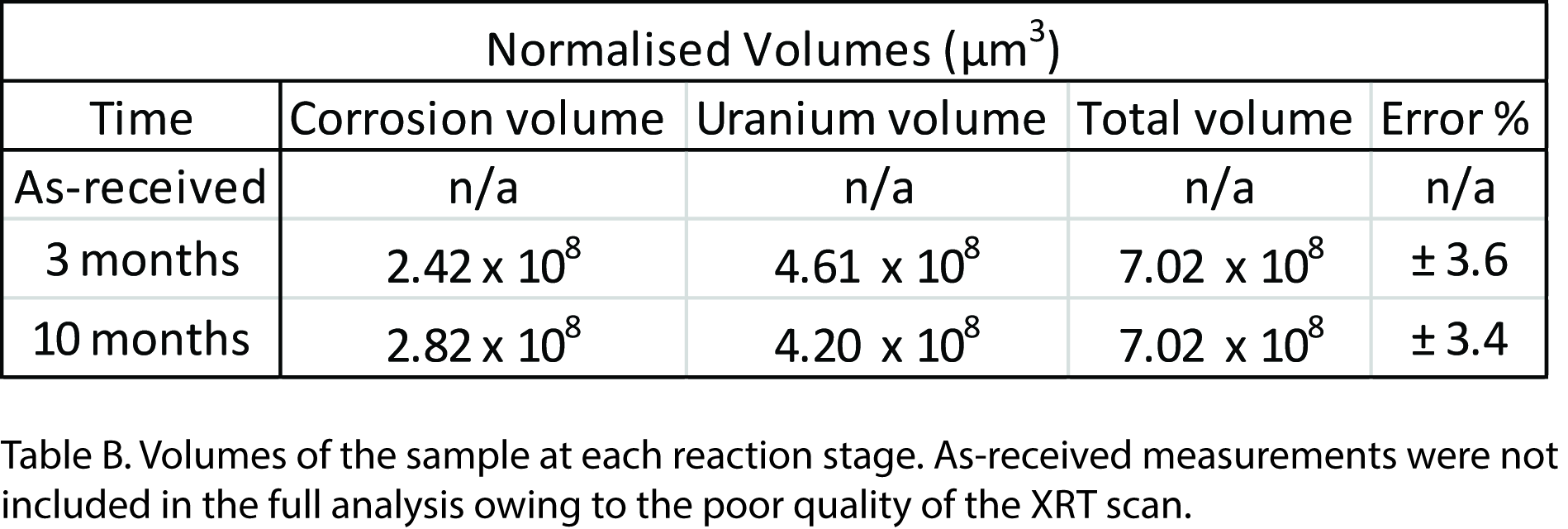

Supplement: S1 Table — Table A. Dimensions of hydrides observed on the sample surface. As-received measurements were not included in the full analysis owing to the poor quality of the XRT scan. Table B. Volumes of the sample at each reaction stage. As-received measurements were not included in the full analysis owing to the poor quality of the XRT scan. Table C. Oxide thicknesses observed on the sample surface at each reaction stage. (ZIP) [file pone.0132284.s003.zip › S1_Table/Table B.tif]

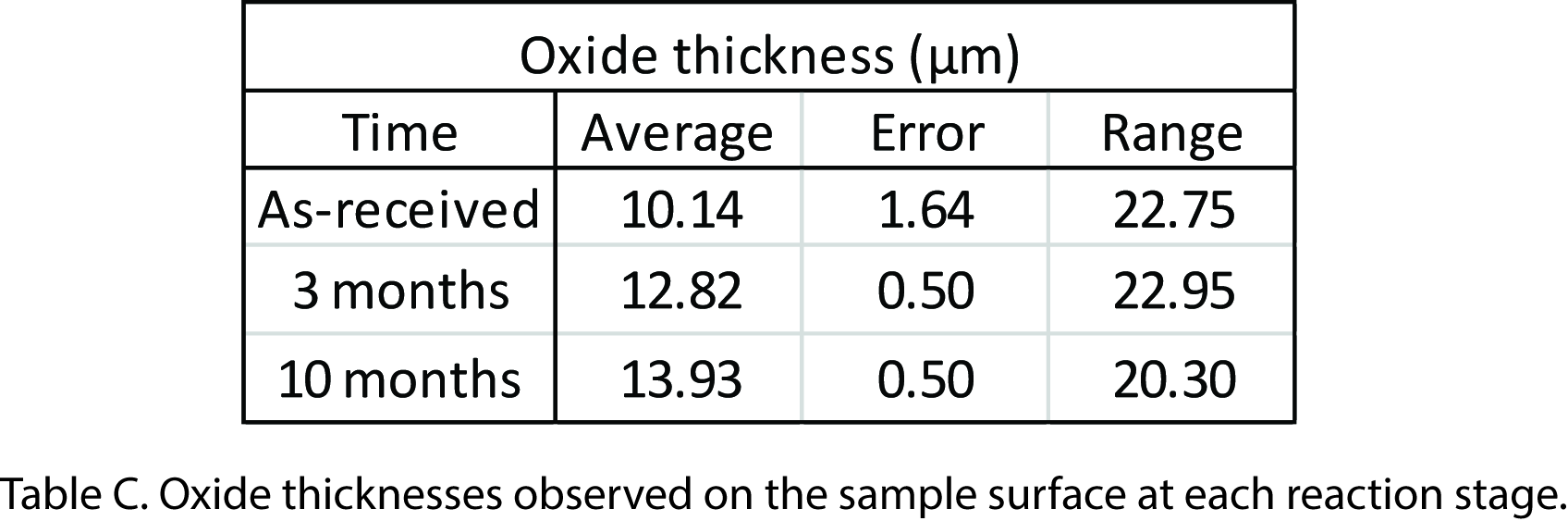

Supplement: S1 Table — Table A. Dimensions of hydrides observed on the sample surface. As-received measurements were not included in the full analysis owing to the poor quality of the XRT scan. Table B. Volumes of the sample at each reaction stage. As-received measurements were not included in the full analysis owing to the poor quality of the XRT scan. Table C. Oxide thicknesses observed on the sample surface at each reaction stage. (ZIP) [file pone.0132284.s003.zip › S1_Table/Table C.tif]
